# Extracranial Trigger Site Surgery for Migraine: A Systematic Review With Meta-Analysis on Elimination of Headache Symptoms

**DOI:** 10.3389/fneur.2019.00089

**Published:** 2019-02-14

**Authors:** Arnaud J. P. E. Vincent, Willem S. van Hoogstraten, Antoinette Maassen Van Den Brink, Joost van Rosmalen, Bibi L. J. Bouwen

**Affiliations:** ^1^Department of Neurosurgery, Erasmus University Medical Center, Rotterdam, Netherlands; ^2^Department of Neuroscience, Erasmus University Medical Center, Rotterdam, Netherlands; ^3^Division of Vascular Medicine and Pharmacology, Department of Internal Medicine, Erasmus University Medical Center, Rotterdam, Netherlands; ^4^Department of Biostatistics, Erasmus University Medical Center, Rotterdam, Netherlands

**Keywords:** migraine, surgery, trigger site, headache, decompression, cauterization

## Abstract

**Introduction:** The headache phase of migraine could in selected cases potentially be treated by surgical decompression of one or more “trigger sites,” located at frontal, temporal, nasal, and occipital sites. This systematic review with subsequent meta-analysis aims at critically evaluating the currently available evidence for the surgical treatment of migraine headache and to determine the effect size of this treatment in a specific patient population.

**Methods:** This study was conducted following the PRISMA guidelines. An online database search was performed. Inclusion was based on studies published between 2000 and March 2018, containing a diagnosis of migraine in compliance with the classification of the International Headache Society. The treatment must consist of one or more surgical procedures involving the extracranial nerves and/or arteries with outcome data available at minimum 6 months.

**Results:** Eight hundred and forty-seven records were identified after duplicates were removed, 44 full text articles were assessed and 14 records were selected for inclusion. A total number of 627 patients were included in the analysis. A proportion of 0.38 of patients (random effects model, 95% CI [0.30–0.46]) experienced elimination of migraine headaches at 6–12 months follow-up. Using data from three randomized controlled trials, the calculated odds ratio for 90–100% elimination of migraine headaches is 21.46 (random effects model, 95% CI [5.64–81.58]) for patients receiving migraine surgery compared to sham or no surgery.

**Conclusions:** Migraine surgery leads to elimination of migraine headaches in 38% of the migraine patients included in this review. However, more elaborate randomized trials are needed with transparent reporting of patient selection, medication use, and surgical procedures and implementing detailed and longer follow-up times.

## Introduction

### Rationale

Migraine is one of the most common diseases worldwide, leading to millions of people taking either preventive or abortive headache medication on a regular basis (1). It also poses a significant economic burden as it is ranked among the most disabling diseases resulting in time lost in the workplace (2, 3). Surgical treatment of migraine headache might be a complementary treatment option for a specific group of migraine patients in which satisfactory pharmacological treatment is not achieved. The surgical treatment comprises decompression of several extracranial branches of the trigeminal and occipital nerve (4) or cauterization of extracranial arteries (5). Although the official neurological diagnosis of migraine describes symptoms far more elaborate than episodes of migrainous headache, (6) this review focuses only on this specific symptom of migraine that is treated with migraine surgery (6). When using the term migraine headache (MH), we thus refer to the headache phase of the complex of migraine symptoms (7). The specific patient group eligible for migraine surgery may consist of patients with identifiable extracranial “trigger sites” where their migraine headache is located. The four identified trigger sites for MH are: frontal, or forehead, region (F), temporal region (T), occipital region (O), and nasal area (N). The exact anatomical features, identification, and surgical approaches have previously been described extensively (8–14).

The concept of treating migraine headache with extracranial surgery finds its roots in early surgical history; (15, 16) in the beginning of the Twentieth century, Oppenheim and Cushing described elimination and changes of migraine symptoms, respectively, following extracranial surgical procedures (17, 18). In the years after, references were made to the possible extracranial origin of certain types of headaches (19) and the possible surgical treatment through extracranial access points (16, 20, 21). Following these initial publications, progress in this research area halted for multiple decades, most likely because of the advent of still improving pharmacological treatment options. In recent years, the number of publications on the surgical treatment of MH has increased, describing new applications of existing plastic surgery procedures (4, 12, 22, 23) in addition to new approaches to the extracranial vasculature (5, 9, 24, 25) and hypotheses about the pathophysiology of migraine and the involvement of extracranial neurovascular regions (26, 27). However, despite several headache clinics applying surgical treatment for migraine, no consensus has been reached concerning the efficacy of migraine surgery. This ambiguity may firstly be due to lacking evidence regarding the exact mechanism of action. It remains puzzling how small perturbations of extracranial microvascular and/or neuronal structures can alleviate or even eliminate symptoms in such a complex disease. Secondly, at least as relevant as the first point, too often studies focusing on migraine surgery base their conclusions on insufficiently documented methodology and results (28). Despite these obvious objections to the use of surgical treatment for a neurological disease, new research is shedding light on processes that might play a role in the pathophysiological background of the disease. Whereas migraine was initially considered as a purely vascular disorder and subsequently thought to be a purely intracranial neurological disorder, it is now clear that migraine is a neurovascular disorder, involving both the brain, nerves and blood vessels (29). Interestingly, more evidence is emerging for the involvement of the extracranial vessels in the pathophysiology of migraine (30, 31).

### Objectives

The objective of this review is to explore whether surgery aimed at disrupting the neurovasculature of extracranial trigger sites is effective for the elimination of migraine headache symptoms in selected migraine patients. Specifically, the proportion of patients reporting elimination of MH after follow-up will be calculated and using data from randomized controlled trials an assessment of the odds ratio for elimination after surgery vs. sham or no surgery will be determined.

### Research Questions

With this study we aim to answer the research question what the proportion of migraine patients is reporting elimination of MH after migraine trigger site surgery and whether surgery compared to sham or no surgery is more effective in the elimination of MH.

## Materials and Methods

### Study Design

We have reviewed all studies published after 2000 and performed a systematic review followed by a meta-analysis to determine the proportion of patients experiencing elimination of migraine headache symptoms. Additionally, we performed a meta-analysis on the included randomized controlled trials (RCTs) to calculate the odds ratio for achieving elimination of headache symptoms in treatment vs. control groups.

### Participants, Interventions, Comparators

Selection of studies was based on in- and exclusion criteria defined prior to the execution of the search. After selection based on title and abstract, the full texts of the remaining studies were reviewed independently by two authors (WH and BB). If both authors agreed, studies were included. In case of discrepancies, a discussion followed. In the case that no agreement was reached, author AV was given the final vote.

**Figure 1:**
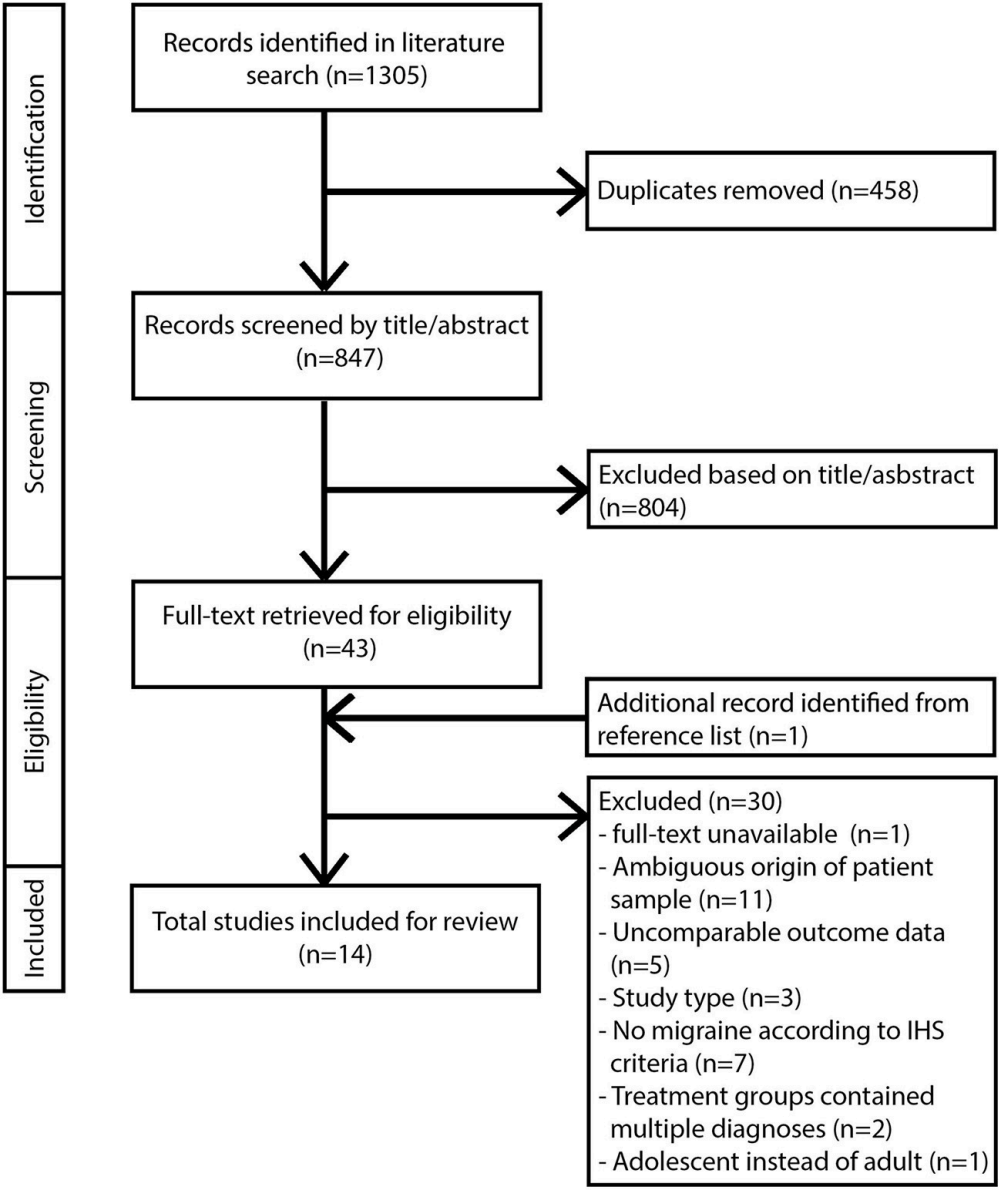
Flow diagram of study selection and reasons for exclusion according to PRISMA guidelines.

Inclusion criteria for this review comprised: studies published between 2000 and March 2018, written in English and performed in adult human subjects. Studies must contain a diagnosis of migraine in compliance with the classification of the International Headache Society (32). For clarification: Behin (33) included patients with typical MH and transformed migraine, a chronic daily headache type with migrainous features that evolves from episodic migraine and has been classified as a sub form of migraine (34). Treatment must consist of one or more surgical procedures involving the extracranial nerves and/or arteries. Outcome data for the treatment groups must be available for a minimum of one time-point with a follow-up of at least 6 months. Appropriate study designs included retro/prospective cohort studies and RCTs. Articles with other study designs were excluded from further analysis. Studies involving radiosurgery, cryosurgery, and any form of nerve stimulation were not included in this review. Additional exclusion criteria for full text articles included missing or unclearly defined study design, patient population, or outcome data. Studies involving treatment with botulinum toxin injections without surgical intervention were excluded. All eligible studies were subsequently evaluated based on their overall methodological quality (35). If the quality of a study was insufficient based on critical appraisal, it was excluded from further analysis. The flow diagram for study selection according to the PRISMA statement guidelines is shown in Figure 1.

### Search Strategy

We performed an online database search on February 17th 2018 combining results from Pubmed, Embase, Medline, Web-of-Science, Cochrane, and Google Scholar. We used search terms (variations used depending on the database syntax) including the following: migraine, surgery, decompression, cauterization, trigger sites. For full search terms per database see Supplementary Table 1. Search results were analyzed and double references were removed. All results were combined in an EndNote (version X7) database. This study was conducted following the PRISMA guidelines for systematic reviews and meta-analyses (36) (see Supplementary Data Sheet 1).

### Data Sources, Studies Sections, and Data Extraction

In migraine surgery literature, outcome assessment is primarily focused on the changes in parameters directly related to the headache phase, i.e., intensity, frequency, and duration. It should be noted that these outcome measures are specific for surgical literature, and that they therefore differ from the common endpoints used in pharmacological migraine studies.

We collected all available data on elimination of MH after migraine surgery at 6 months to 1-year follow-up. The percentage of patients with elimination of headaches after surgery was taken from the articles or, if necessary, calculated *post hoc* based on patient numbers and outcome data. If a study applied multiple surgical approaches and performed a qualitative comparison of procedures (i.e., scopic vs. open approach), we collected outcome data from both surgical treatment groups.

For the definition of MH elimination, we applied the following criteria: 100% elimination of migraine headache intensity, duration and frequency at follow-up from baseline values. This definition was derived from the measures applied in the surgical migraine literature. The elimination group was compared to patients experiencing ≤ 99% improvement of the three measures mentioned above at follow-up. Using these outcomes, we determined the proportion of patients who experienced elimination of symptoms at follow-up.

All included studies except for one reported outcome for the elimination group as 100% elimination of symptoms at follow-up. The only study reporting an alternate definition of elimination was Dirnberger and Becker (37), reporting elimination as 90–100% elimination of headache days. However, since they report their results as an absolute reduction of headache days and not a combination of frequency, intensity, and duration of MH (also known as migraine headache index (MHI) score which is generally considered to be a less precise measure than absolute number of headache days), this study was included in the analysis.

For the meta-analysis of the odds-ratio for elimination (reported in **Figure 3**), all articles selected patients eligible for surgery based on the response to botulinum toxin injection at the designated trigger sites. Trigger sites were defined as responsive if MH symptoms at the location reduced at least 50% in frequency, intensity or duration in response to the botulinum toxin injection. Although selection with botulinum toxin is not a current clinically used diagnostic tool or treatment modality for migraine, it is a common selection feature of migraine surgery studies and is seen as an indication for treatment effectivity (38).

### Data Reporting and Selection

**Figure 2:**
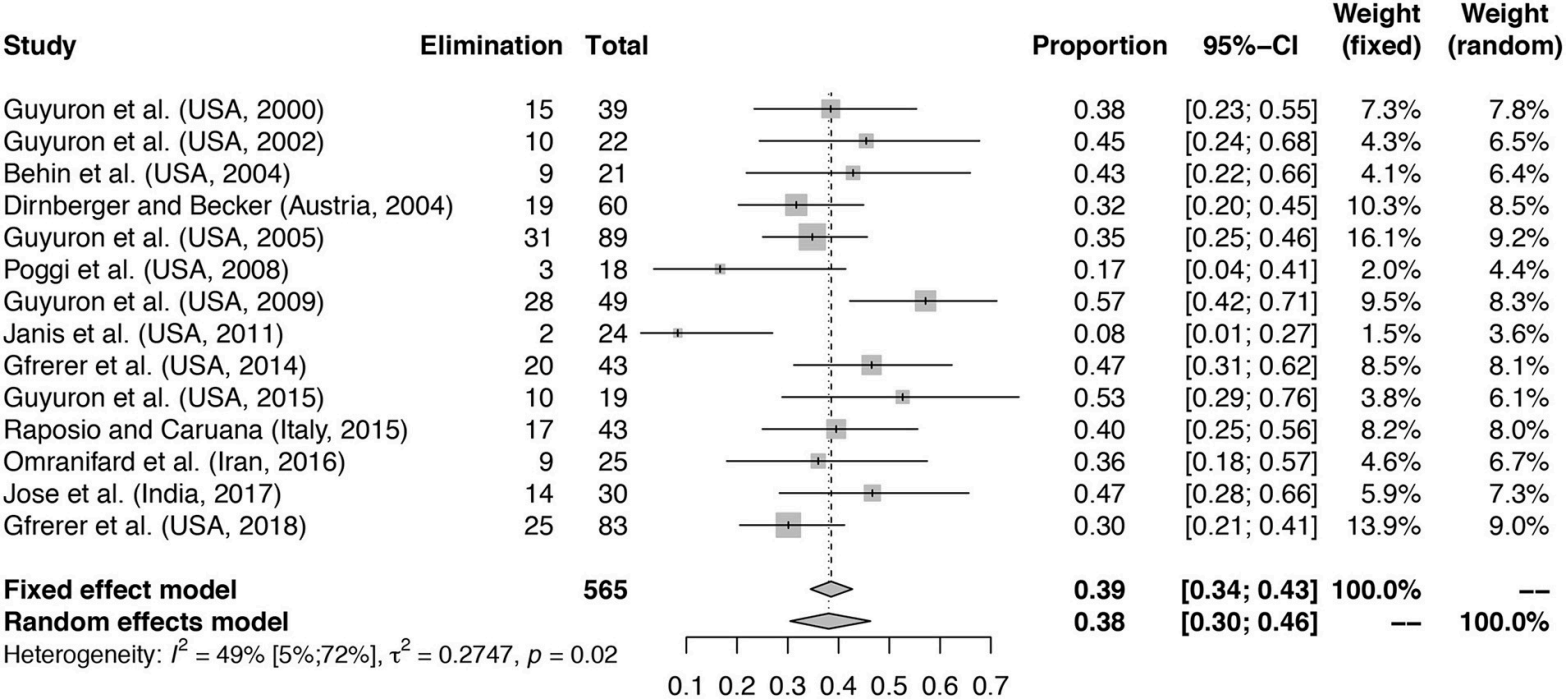
Proportion of patients with elimination of MH at follow-up. Meta-analysis with forest plot of the proportions of patients with elimination of MH after migraine surgery at follow-up. Study column reports author, country and year of publication. The total number of treated patients per study is shown with the number of patients reporting elimination at follow-up. Elimination column reports total number of patients with elimination of MH at follow-up. Total is the total number of patients included in the surgical arm of the study. The proportion column represents the proportion patients with elimination of symptoms with 95% confidence interval (95%-CI) with fixed and random effects model weights. Mean proportion for fixed effects model and random effects model with confidence interval given. Heterogeneity calculations based on the Hartung–Knapp–Sidik–Jonkman estimator with *p*-value based on Cochran's Q given.

The heterogeneity in data and outcome reporting was high between studies. With the exception of Janis et al. (39), Raposio et al. (12), Guyuron et al. (40), and Jose et al. (41), studies did not report in detail whether unilateral or bilateral procedures were performed, therefore no further analysis based on laterality of the procedure was made. Six out of 14 studies report performing surgery on three or more surgical sites, among which are the three RCTs. In some studies, patients could receive surgery on multiple trigger sites during the same procedure (42–45). The outcome data were in those cases pooled per patient without reporting elimination scores for separate sites. The pooling of this data prohibited any analysis of outcome per individual trigger site, therefore this was not analyzed further. The study of Guyuron et al. (40) compares two surgical approaches for temporal trigger site surgery, performing both procedures in all patients. In order to ascertain data and outcomes per patient instead of per site, as this study reported, we chose to only analyse the decompression surgery data. For Gfrerer et al. (46), 8 of 35 patients received a second trigger site procedure after their initial trigger site operation. Outcome at 1-year follow-up was subsequently analyzed for the 43 trigger sites operated and was not reported per patient individually. These data are therefore reported as the total number and number of eliminations for the sites in **Table 2** and in Figure 2.

### Data Analysis

Two separate meta-analyses were performed. For the meta-analysis of the proportion of patients with elimination of symptoms at follow-up, a random effects model with a logit transformation was used to compute the pooled proportion and associated 95% CI. For individual studies, 95% binomial proportion CIs were calculated using the Clopper-Pearson method. RCT and cohort study data was combined to assess the general percentage of patients reporting elimination after surgery (48). For the meta-analysis of the treatment effect of migraine surgery in the included randomized controlled trials, a random effects model for the odds ratio of migraine surgery was used to compute the pooled odds ratio and associated 95% CI. A random effects model was chosen over a fixed effects model because heterogeneity between studies was expected (49). The treatment effect of migraine surgery was analyzed by comparing the proportion of patients with complete elimination of MH after surgery (calculated as the number of patients experiencing elimination divided by the total number of patients in the group) to the proportion of patients experiencing elimination of symptoms in the control group resulting in the odds ratio for elimination of MH symptoms after surgery. In both meta-analyses, the Hartung–Knapp–Sidik–Jonkman method was applied to estimate the between-study variance, and a continuity correction of 0.5 was applied in case of studies with zero cell frequencies. The heterogeneity of combined study results was assessed using the inconsistency statistic (*I*^2^ and confidence interval of *I*^2^) and tested using Cochran's Q test. For assessment of publication bias, funnel plots were generated. The meta-analyses were performed using R version 3.4.3 and the R packages *meta* and *metafor*.

## Results

### Study Selection and Characteristics

We identified 847 unique records from six different databases after removal of duplicates. All identified records were screened for eligibility by title and abstract. Forty-four full text articles were reviewed for inclusion. Fourteen studies satisfied all in- and exclusion criteria and are included in this review (Figure 1).

The general characteristics of the included studies are summarized in Table 1. A total of 627 patients are included in this review including treatment and control groups. The sample size varied from 18 to 108 patients and follow-up period ranged from 6 months in three studies (12, 33, 37) to 1 year or more in the other studies. The age of study participants ranged from 18 to 80 years and percentage of male participants varied between 2.6 and 27.5%. Surgical approaches to the four distinct trigger sites were either through an open approach with skin incision, or via endoscopic approach, also used for cosmetic surgical applications.

**Table 1:** Characteristics of included studies.

**Authors (country, year of publication)**	**Sample size (N) | mean age [range] (years)**	**% Male**	**Follow-up (months)**	**Study design**	**Migraine subtype**	**Trigger sites**	**Surgical approach**
Guyuron et al. (USA, 2000) (4)	39 | 56 [32-70]	2.6	47 [5-122]	Retrospective cohort	Migraine	F	Skin incision/endoscopic/open forehead approach
Guyuron et al. (USA, 2002) (14)	22 | 44.9 [24-58]	18.2	12 [7-16]	Prospective cohort	Migraine (moderate–severe)	F and T	Skin incision/endoscopic
Behin (USA, 2004) (33)	21 | 45 [21-73]	27.5	6–62	Retrospective cohort	Migraine and transformed migraine	N	Endoscopic
Dirnberger and Becker (Austria, 2004) (37)	60 | [24-80]	21.7	6 [6-18]	Prospective cohort	Migraine	F	Skin incision
Guyuron et al. (USA, 2005) (42)	108 | 43.4 ± 1.1	Unknown	12 [7-21]	RCT	Migraine (moderate—severe)	F, T, O, and N	Skin incision/endoscopic
Poggi et al. (USA, 2008) (45)	18 | 41 [22-53]	11	16 [6-41]	Retrospective cohort	Migraine	F, T and O	Skin incision/endoscopic
Guyuron et al. (USA, 2009) (43)	75 | 44.9 [26-76]	Unknown	12	RCT	Migraine (moderate - severe)	F, T, and O	Skin incision/endoscopic
Janis et al. (USA, 2011) (39)	24 | 44.4 [23-66]	4.2	22 [5-53]	Retrospective cohort	Migraine	F, T, O, and N	Skin incision/endoscopic
Gfrerer et al. (USA, 2014) (46)	35 | 46.1 [20-72]	14.3	12	Prospective cohort	Chronic refractory migraine	F and T	Skin incision
Guyuron et al. (USA, 2015) (40)	19 | 38.2 [19-62]	5.3	12	prospective cohort	Migraine	T	Skin incision
Raposio and Caruana (Italy, 2015) (12)	43 | [18-72]	11.6	6	Prospective cohort	Migraine	F	Endoscopic
Omranifard et al. (Iran, 2016) (44)	50 | 42.2 ± 6.9	12	12 [11-15]	RCT	Migraine	F, T, O, and N	Skin incision/endoscopic
Jose et al. (India, 2017) (41)	30 | 36.4 ± 9.2	23.3	11.1	Prospective cohort	Migraine	F and T	Skin incision
Gfrerer et al. (USA, 2018) (47)	83 | 45 [18-73]	14	12.3	Prospective cohort	Chronic refractory migraine	F, T, O, and N	Skin incision

*The table shows an overview of all included studies ordered by year of publication. Author(s), country, and year of publication are reported. Total sample size and mean age (if available) with range (if reported) are in absolute numbers. Percentage of male participants is given when reported. Follow-up at time of outcome measurement was recalculated into months where necessary, range is given if known. Specific migraine subtype described in the original article is given. Trigger sites are summarized, one or multiple trigger sites listed. Description of surgical approach used in the study is summarized in the last column, for specific details on approach we refer to the respective articles. RCT, randomized controlled trial; F, Frontal; T, temporal; O, Occipital; N, Nasal*.

#### Elimination of Migraine Symptoms at 6–12 Month Follow-Up

A total number of 565 patients treated with migraine surgery was included in the analysis, a summary is given in Table 2. Figure 2 shows that a proportion of 0.38 of patients (random effects model, 95% confidence interval [0.30–0.46], heterogeneity: *I*^2^ = 49% [5%; 72%], *p* = 0.02) reported elimination of MH at follow-up. The significant *p*-value for *I*^2^ suggests heterogeneity of the data. This heterogeneity is mainly due to two outliers with stricter handling of the definition of elimination, namely Janis et al. (39) and Poggi et al. (45). Meta-analysis with exclusion of these studies led to an *I*-squared value of 26% [0%; 62%], *p* = 0.19 and a proportion of elimination of 0.41 [0.35; 0.46] (Random-effects model, forest plot not shown, funnel plot shown in Supplementary Figure 1B). A second sensitivity analysis excluding the RCTs from the meta-analysis led to a proportion of 0.38 [0.29; 0.49] of patients with elimination of MH (Random effects model, *I*^2^ = 54.0% [9.1%; 76.7%], *p* = 0.02, forest plot not shown). Again, the I-squared value is dependent on the two outliers formed by Poggi et al. (45) and Janis et al. (39).

**Table 2:** Outcome after migraine surgery.

**Author(s) (country, year of publication)**	***N* treated**	**Elimination (%)**	**Improvement (%)**	**No improvement (%)**
Guyuron et al. (USA, 2000) (4)	39	15 (38.5)	16 (41)	8 (20.5)
Guyuron et al. (USA, 2002) (14)	22	10 (45.5)	11 (50)	1 (4.5)
Behin (USA, 2004) (33)	21	9 (42.9)	7 (33.3)	5 (23.8)
Dirnberger and Becker (Austria, 2004) (37)	60	19 (31.7)	16 (26.7)	25 (41.7)
Guyuron et al. (USA, 2005) (42)	89	31 (35)	51 (57)	7 (8)
Poggi et al. (USA, 2008) (45)	18	3 (16.7)	13 (72.2)	2 (11.1)
Guyuron et al. (USA, 2009) (43)	49	28 (57.1)	13 (26.5)	8 (16.3)
Janis et al. (USA, 2011) (39)	24	2 (8.3)	17 (70.8)	5 (20.8)
Gfrerer et al. (USA, 2014) (46)	43 SITES	20 (46.5)	19 (44.2)	4 (9.3)
Guyuron et al. (USA, 2015) (40)	19	10 (52.6)	6 (31.6)	3 (15.8)
Raposio and Caruana (Italy, 2015) (12)	43	17 (39.5)	18 (41.9)	8 (18.6)
Omranifard et al. (Iran, 2016) (44)	25	9 (36.0)	10 (40)	6 (24)
Jose et al. (India, 2017) (41)	30	14 (46.7)	14 (46.7)	2 (6.6)
Gfrerer et al. (USA, 2018) (47)	83	25 (30.0)	43 (52)	15 (18)

*Outcome data at follow-up is summarized in this table. In the first column, the number of patients or surgery sites treated with migraine surgery in the study is reported. The second column states the number of patients with elimination of migraine headache with percentage of total treated patients between brackets. Gfrerer et al. (46) is reported in number of surgical sites because of lacking individual patient outcomes. The third column reports patients with improvement of symptoms defined as 50–99% improvement of migraine headache symptoms. Patients reporting < 50% or no improvement are reported in the last column*.

With regard to the mean proportion of elimination, Janis et al. (39) and Poggi et al. (45) report lower elimination rates compared to the others. However, both studies report substantially higher percentages of patients having 50–99% improvement compared to the other included studies (Table 2), which might be due to a stricter handling of the definitions of elimination and strong improvement and lead to lower elimination proportions. Poggi et al. (45) included a group of 75–99% improvement and 50% of the patients ended in this group at follow-up. Janis et al. (39) show an elimination percentage per individually operated site ranging from 35% (frontal region) to 62.5% (nasal region), however some patients did not reach elimination in all trigger sites, which might influence the final number of patients with full elimination. The number of patients reporting < 50% improvement in both studies is similar to that of the other included studies. The studies of Guyuron et al. (43) and Guyuron et al. (40) have a high elimination rate compared to the others. One aspect in Guyuron et al. (43) that might contribute to this is that patients only received one surgery on their most prevalent site of migraine headache and not receive surgery for other trigger sites. Thus, the elimination rates were reported after surgery for that specific site. What is not reported, however, is whether these patients still had migraine originating from other trigger sites, which could mean that in some patients the migraine headaches did not fully disappear but for the mentioned trigger site. Guyuron et al. (40) compared two surgical approaches at the temporal site and showed for both approaches a high percentage of elimination. This could either be due to selection of the patients or perhaps to a high chance of success of this particular surgical site.

#### Migraine Surgery vs. Sham or no Surgery at 1-Year Follow-Up

Three studies performed an RCT comparing surgical treatment to sham or no surgery, with several notable differences in methodology. Guyuron et al. (43) compared migraine surgery to sham surgery; patients were randomly assigned to treatment groups in a two-third (treatment) vs. one-third (sham) ratio and patients and investigators (independent of the surgical team) were blinded to their treatment group until data collection was finalized. Omranifard et al. (44), randomly assigned patients in a 1 to 1 ratio and the control group did not receive surgery but continued standard therapy with medication. Guyuron et al. (42) randomly assigned patients in a 4 to 1 ratio; control patients were given a saline injection and did not receive sham surgery, no data on continued pharmacological treatment during follow-up period in control patients was given.

**Figure 3:**
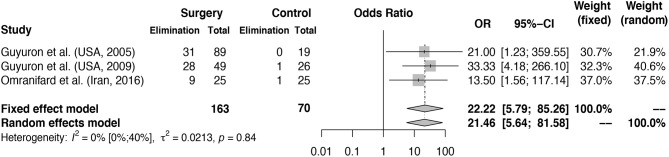
Odds ratio for elimination of MH symptoms at 1 year follow-up: intervention vs. control groups. Meta-analysis of three RCTs reporting MH elimination outcomes at 1 year is shown in the figure. Odds ratio for elimination of symptoms in surgery group vs. control group calculated. The surgery column represents the number of patients with elimination after surgery and the control column represents the number of patients with elimination of MH in the control groups. The total column reports the total number of patients in the surgery and control groups. Fixed and random effects model final odds ratio is given. OR, odds ratio. 95%-CI, confidence interval. Heterogeneity calculations based on the Hartung–Knapp–Sidik–Jonkman estimator with *p*-value based on Cochran's Q given.

The calculated odds ratio for elimination of migraine headache at 1-year follow-up is 21.46 (random effects model, 95% CI [5.64–81.58], heterogeneity: *I*^2^ = 0% [0%; 62%], *p* = 0.8396) for patients receiving migraine surgery compared to sham or no surgery, shown in Figure 3. Of the 163 patients receiving surgery, 68 (41.7%) reported elimination after 1 year. Of the 70 control patients, only two patients reported elimination after sham or no surgery. Of the 163 patients receiving surgical treatment, 21 (12.5%) reported ≤ 50% improvement of symptoms, considered to be “no improvement,” compared to 52.8% (37 of 70) of the control patients. In the study of Guyuron et al. (43), a percentage of 57.7% of patients reported significant improvement in the sham group compared to 83.7% in the treatment group. This high percentage of improvement in the sham group was not further explained but could be partly due to a rather large placebo effect. In Guyuron et al. (42) the percentage of improvement in the sham group was 15.8% (3 of 19 patients). In the study of Omranifard et al. (44) 40% (10 out of 25) of patients in the control group (which received only medication treatment) experienced improvement.

#### Adverse Events

Several studies reported data on adverse events after surgery (full list shown in Supplementary Table 2). The most frequent general adverse events are temporary swelling, numbness, or shooting pain postoperatively. Less frequent (25–75%) are temporal hollowing (for the temporal trigger site), immediate postoperative headache, alopecia and (temporary) ecchymosis.

The most frequent adverse event for the temporal trigger site found in Guyuron et al. (43) is temporal hollowing in 53% of patients. Guyuron et al. (14) reported temporal numbness in all patients for a period ranging from 1 to 6 months after surgery, with an average of 2.3 months. In all patients the numbness recovered. At the nasal trigger site, problems with nasal dryness or rhinorrhoea and partial recurrence of septal deviation were reported in Guyuron et al. (42). For the frontal trigger site the most frequently reported adverse events are itching and hair loss as reported by Guyuron et al. (42) and Gfrerer et al. (46). At the occipital trigger point neck stiffness (42) and numbness (46) were the most common adverse events. At 1-year follow-up the majority of the listed adverse events had resolved. No major adverse events were reported.

#### Assessment of Publication Bias

To assess whether the meta-analysis in Figure 2 suffers from publication bias, we rendered funnel plots, see Supplementary Figure 1. For the elimination of migraine symptoms at 6–12 months follow-up, all included studies are seemingly randomly dispersed along the funnel axis, with the exception of Janis et al. (39) and Poggi et al. (45), and there is no clear indication of publication bias. However, as described previously these studies reported a smaller proportion of patients with elimination, most likely due to a stricter definition of elimination of MH (Supplementary Figure 1B, outliers excluded). Due to the limited number of studies in the meta-analysis of the odds ratio for elimination, no funnel plot was generated.

## Discussion

### Summary of Main Findings

In this systematic review and meta-analysis of migraine trigger site surgery, we demonstrate that in selected migraine patients with identifiable extracranial trigger points, the odds ratio for elimination of migraine headache after surgery is 21.46 for surgically treated patients compared to controls. Also, we report that the average proportion of patients having elimination of MH post-surgery is 38% after 6–12 months follow-up. In the remaining proportion of patients that do not experience full elimination of headaches, there was still a substantial proportion that reported 50–99% reduction in duration, intensity, and/or frequency of their headaches. Logically, a significant reduction of MH (but no elimination) can still lead to considerably less use of migraine medication and higher quality of life. However, the documentation of quality of life after migraine surgery is scarce and has been reported by few groups (5, 42, 50). Given the results of this study, it is an important factor that should be considered by future studies investigating the effects of surgery.

### Interpretation of Results and Limitations

The endpoints used in migraine surgery trials should be seen in a separate light from those used in pharmacological migraine trials. In migraine surgery literature, outcomes are limited to symptoms related the headache phase. There is currently no data available describing possible changes in character of the MH after surgery, which could potentially be an important remaining factor post-surgery. If migraine surgery cannot fully eliminate the headaches for a patient, but the MH changes to a more benign form, quality of life could still be significantly improved. The included studies are often performed in (mostly female) patients that have tried many different pharmacological treatments with unsatisfying result. As a result of this, it is likely that many patients are prone to medication overuse. A placebo effect can therefore not be excluded, which also shows from the high percentage of patients in the sham groups reporting significant improvement of their MH. The ethical aspects of performing sham surgery in a randomized surgical trial make this a challenging but important factor in studying the effectivity of migraine surgery.

Another important factor in this meta-analysis to consider is the total number of included studies and patients. Low numbers inevitably alter the reliability of subsequent analyses and generalizability of found results. However, analysis of confidence intervals of individual studies included in the analyses was performed to assess heterogeneity and outliers, as were several sensitivity analyses to account for the heterogeneity of data. Especially for the second meta-analysis, caution should be taken to interpret the calculated odds ratio, given the small *I*^2^ value and the broad confidence interval for *I*^2^. This value is due to the low number of studies that could be included, and should in this case be interpreted carefully because of risk of bias (51). Furthermore, regression to the mean is an important effect that occurs in headache research and cohort studies, especially when recording pain scores (52). However, considering the relatively long follow-up time used and the high response rate of study participants, placebo effect and regression to the mean, although they can and should not be discarded, are unlikely to be solely responsible for the treatment effect observed in the prospective cohorts and randomized trials.

The evidence from the included studies should be viewed with caution due to limitations in methodology and risk of multiple forms of bias, including, but not limited to, selection and observer bias. An important limitation of the included surgical articles is a lack of transparent patient selection methods with the obvious risks of selection bias and lack of detailed patient characteristics before/after surgery. There is often absence of clearly defined and widely-used outcome measures and accurate description of pharmacological treatment during the full study period. Furthermore, the lack of clear definitions for the sham groups and their (however logical due to ethical considerations) small group size with uneven allocation of patients to treatment and control groups leads to a considerable expectancy effect in the sham groups.

However, despite these clear limitations there is a pressing need for evaluation of these methods, as migraine surgery is currently being carried out in a several practices worldwide. This highlights the necessity for this review, providing a report of the current standings in literature, and also urges the start of new well-performed and documented randomized controlled trials in the future.

Interestingly for example, based on this analysis, compared to other non-pharmacological treatments for MH such as transcranial magnetic stimulation (TMS) or transcutaneous supraorbital nerve stimulation (t-SNS), the effect size of surgery is comparable if not larger (53, 54). The important advantage of surgery over transcranial stimulation approaches, however, is that patients need to undergo a single episode of surgical intervention compared to the long-term use of stimulators. Additionally, an ethical argument for the further study of headache surgery can be made. In cluster headache, various surgical approaches are used as a last resort treatment option when pharmaceutical treatment proves insufficient to relieve headache symptoms (55). Combined with the results in this study, the use of surgery and its effectivity for cluster headache and potentially also migraine headaches may form a basis for more research into these new applications.

For this meta-analysis, we chose to analyse studies consisting only of migraine patients undergoing specific surgical treatment. We selected migraine headache (excluding other headache types such as cluster headache or (chronic) tension type headache) as a main focus, in an attempt to demarcate our analysis to a relatively well-defined population. Furthermore, we only analyzed the groups with full elimination of MH. This is because many studies use the migraine headache index (MHI) as their surgical outcome measurement tool, which is a much-criticized scoring system for migraine headaches making other categories such as improvement after surgery hard to assess (28). To improve the reporting of migraine surgery effects, future studies should focus instead on describing reduction in headache days and pain scores for the remaining headache days.

As a result of the factors stated above, the reported odds ratio and proportion of elimination are only applicable in a small group of patients. This limitation is outweighed by the methodological advantages and the higher reliability of the meta-analysis. This is an important consideration, since the field of migraine surgery is plagued by many controversies regarding variation in surgical techniques, description of methodology and patient selection, as previously stated.

As many reviewers and headache surgery critics have previously described, poor documentation of these aforementioned elements is an important limitation that must be considered when interpreting the data (28, 56–58). Furthermore, as an example of lacking comparing outcome data, we were forced to exclude the study of Shevel (5) in the selection process, which is one of the few studies that describes cauterization of the extracranial arteries and not decompression as a surgical approach for MH (5). Interestingly however, a recently published randomized cohort study from the Guyuron group also argues that arterectomy in combination with decompression of the forehead trigger site, gave improved outcomes compared to the same procedure without arterectomy (59). This adds to the pathophysiological question which of the structures located at the trigger site is the key structure to address and the most likely to cause relief of the migrainous headache phase (60).

## Conclusions

Based on the currently available evidence included in this review we would cautiously conclude that a subset of selected migraine patients with distinctly identifiable extracranial MH trigger sites may benefit from surgical decompression of the neurovasculature. Important to state is that only a minority of migraine patients with specific trigger sites may benefit from this treatment and that current selection procedures for treatment need optimization and further investigation. Patients are now often being selected with botulinum toxin in an out-of-standard dosage. Since migraine surgery is already being employed by some clinics, albeit on small scale, it is important for both physicians and patients to critically appraise this treatment and its effectivity. Standardization of surgical approaches is of key importance for a further assessment of the real effect of this treatment (61). Therefore, we urge researchers in the field to give migraine surgery research a critical but simultaneously open-minded appraisal and encourage other groups to study this approach in more detail to ultimately aid in finding better treatment solutions for the long-time suffering migraine patient.

## Author Contributions

BB and AV designed and conceptualized the study. BB and WvH performed database searches and performed the systematic review of articles, both also analyzed the data. BB drafted the manuscript for intellectual content. JvR designed the meta-analysis and wrote the statistics section of the manuscript. AM and AV co-wrote the manuscript, interpreted data, and revised the manuscript for intellectual content. All authors read and approved the final manuscript. AV and BB share the correspondence for the manuscript.

### Conflict of Interest Statement

The authors declare that the research was conducted in the absence of any commercial or financial relationships that could be construed as a potential conflict of interest.

## Supplementary Material

Supplementary Figure 1 Funnel plots for assessment of publication bias. **(A)** Funnel plot for Figure 2-Proportion of patients with elimination of MH at follow-up. X-axis represents the logit transformed proportion of patients with elimination of headache for each included study. Y-axis represents standard error of study based on number of included study participants. Outliers can be seen on the left-hand side of the plot, representing a very low proportion of elimination. **(B)** Funnel plot for Figure 2, but with exclusion of two outliers as described in Results section. Note the narrower area of the funnel due to the exclusion of two outliers.

Supplementary Table 1 Overview of search terms used per database. Database search performed on February 17th 2018.

Supplementary Table 2 Adverse events. Adverse events reported based on their prevalence reported in the included studies. Superscripted numbers refer to the respective studies at the bottom of the table. Small numbering used for respective studies, studies reported as author (year of publication). F, frontal; T, temporal; N, nasal; O, occipital.

Supplementary Data Sheet 1 PRISMA 2009 Checklist.
