# Supplementary figures and images for: Extracranial Trigger Site Surgery for Migraine: A Systematic Review With Meta-Analysis on Elimination of Headache Symptoms
Source: Front Neurol. 2019 Feb 14;10:89. doi: 10.3389/fneur.2019.00089 (PMC6383414; doi:10.3389/fneur.2019.00089)

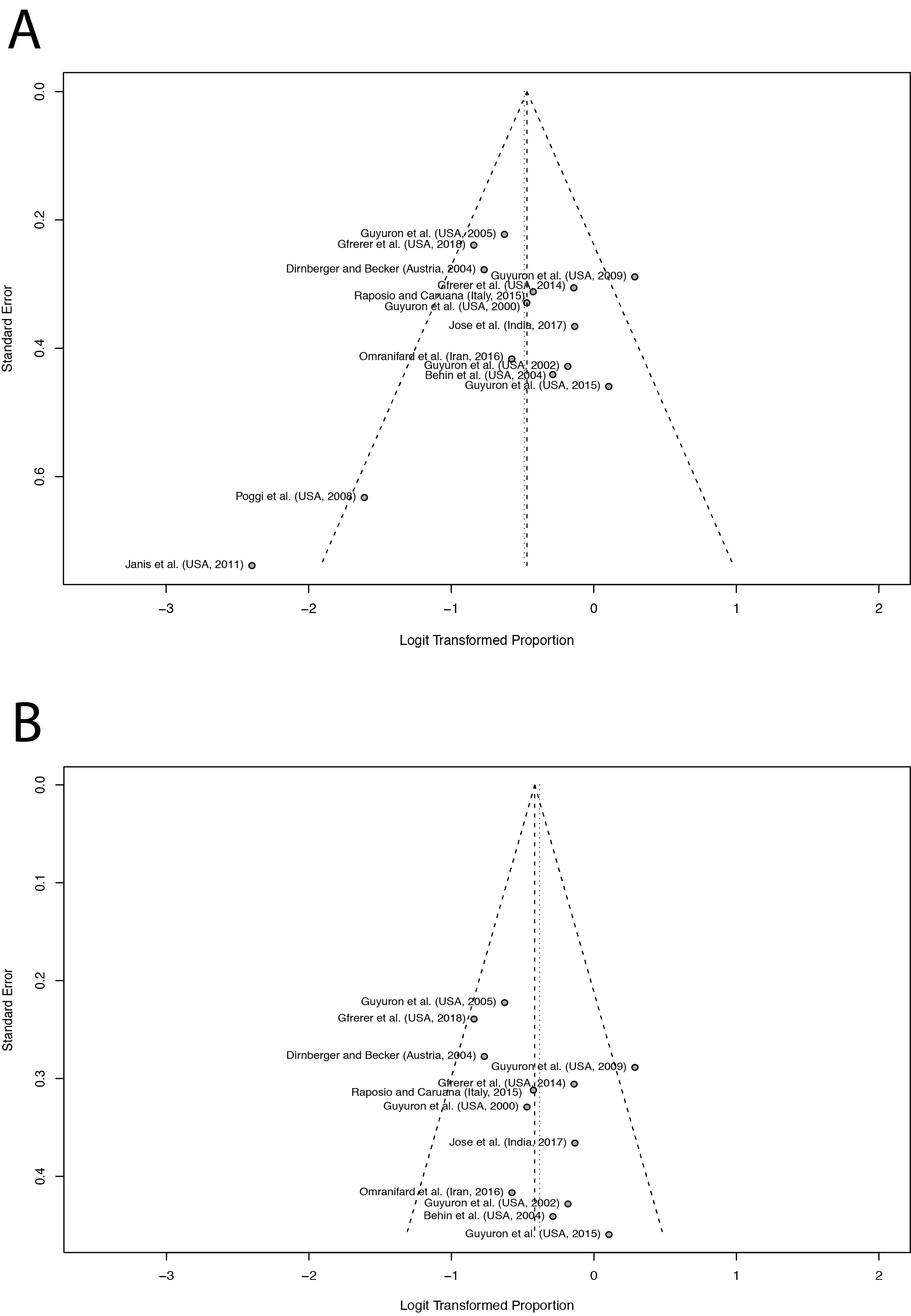

Supplement: Supplementary Figure 1 — Funnel plots for assessment of publication bias. (A) Funnel plot for Figure 2-Proportion of patients with elimination of MH at follow-up. X-axis represents the logit transformed proportion of patients with elimination of headache for each included study. Y-axis represents standard error of study based on number of included study participants. Outliers can be seen on the left-hand side of the plot, representing a very low proportion of elimination. (B) Funnel plot for Figure 2, but with exclusion of two outliers as described in Results section. Note the narrower area of the funnel due to the exclusion of two outliers. [file Image_1.PNG]
