# Supplementary material for: Extracranial Trigger Site Surgery for Migraine: A Systematic Review With Meta-Analysis on Elimination of Headache Symptoms
Source: Front Neurol. 2019 Feb 14;10:89. doi: 10.3389/fneur.2019.00089 (PMC6383414; doi:10.3389/fneur.2019.00089)
Supplement: Supplementary Table 1 — Overview of search terms used per database. Database search performed on February 17th 2018. [file Table_1.DOCX]

Supplementary Material

**Extracranial trigger site surgery for migraine: a systematic review with meta-analysis on elimination of headache symptoms**

**Ms Bibi L.J. Bouwen^1,2,†^, MSc, Mr Willem S. van Hoogstraten^2,†^, BSc, Mrs Antoinette Maassen Van Den Brink^3^, PhD, Mr Joost van Rosmalen^4^, Mr Arnaud J.P.E. Vincent^1*^, MD PhD**

***Corresponding author**

dr. Arnaud J.P.E. Vincent, MD PhD, neurosurgeon

Email: [a.vincent@erasmusmc.nl](mailto:a.vincent@erasmusmc.nl)

Telephone number: +31 10 70 34 211

Address: Erasmus University Medical Center, PO Box 2040, 3000 CA, Rotterdam, the Netherlands

# Supplementary Data

**Supplementary Table 1 – Overview of search terms used per database**

Database search performed on February 17^th^ 2018.

**Supplementary Table 2 – Adverse Events**

Adverse events reported based on their prevalence reported in the included studies. Superscripted numbers refer to the respective studies at the bottom of the table. Small numbering used for respective studies, studies reported as author (year of publication).Abbreviations: F = frontal, T = temporal, N = nasal, O = occipital.

**Supplementary Table 1**

| Database | Search terms used |
| --- | --- |
| Embase.com | (migraine/exp OR (migrain* ):ab,ti) AND (&#39;artery surgery&#39;/de OR &#39;artery ligation&#39;/de OR  &#39;microvascular decompression&#39;/exp OR &#39;muscle denervation&#39;/exp OR &#39;headache and facial  pain&#39;/exp/dm_su OR cauterization/exp OR electrosurgery/exp OR &#39;catheter ablation&#39;/exp OR  (((microvascular*) NEAR/3 (decompress*)) OR (muscle* NEAR/3 denervat*) OR cauter* OR ((&#39;trigger  site&#39; OR &#39;trigger sites&#39; OR surg* OR operat*) NEAR/6 deactivat*) OR ((arter* OR endonasal*) NEAR/3  (surg* OR ligation* OR operat*)) OR electrosurg* OR ((migraine* OR headache*) NEAR/3 (surg* OR  cryosurg* OR neurosurg* OR operat*))):ab,ti) |
| Medline (OvidSP) | (exp Migraine Disorders/ OR (migrain*).ab,ti.) AND (exp arteries/su OR exp Migraine Disorders/su  OR exp Headache/su OR exp cautery/ OR (((microvascular*) ADJ3 (decompress*)) OR (muscle* ADJ3  denervat*) OR cauter* OR ((trigger site OR trigger sites OR surg* OR operat*) ADJ6 deactivat*) OR  ((arter* OR endonasal*) ADJ3 (surg* OR ligation* OR operat*)) OR electrosurg* OR ((migraine* OR  headache*) ADJ3 (surg* OR cryosurg* OR neurosurg* OR operat*))).ab,ti.) |
| Cochrane | ((migrain* ):ab,ti) AND ((((microvascular*) NEAR/3 (decompress*)) OR (muscle* NEAR/3 denervat*)  OR cauter* OR ((&#39;trigger site&#39; OR &#39;trigger sites&#39; OR surg* OR operat*) NEAR/6 deactivat*) OR ((arter*  OR endonasal*) NEAR/3 (surg* OR ligation* OR operat*)) OR electrosurg* OR ((migraine* OR  headache*) NEAR/3 (surg* OR cryosurg* OR neurosurg* OR operat*))):ab,ti) |
| Web-of- science | TS=(((migrain*)) AND ((((microvascular*) NEAR/3 (decompress*)) OR (muscle* NEAR/3 denervat*)  OR cauter* OR ((&quot;trigger site&quot; OR &quot;trigger sites&quot; OR surg* OR operat*) NEAR/6 deactivat*) OR  ((arter* OR endonasal*) NEAR/3 (surg* OR ligation* OR operat*)) OR electrosurg* OR ((migraine*  OR headache*) NEAR/3 (surg* OR cryosurg* OR neurosurg* OR operat*))))) |
| Google scholar | Migraine surgery\|surgical\|surgeries\|&quot;microvascular decompression&quot;\|&quot;muscle  denervation&quot;\|cautery\|cauteriozation\|&quot;trigger*deactivation&quot;\|&quot;arterial surgery&quot;\|electrosurgery |

**Supplementary Table 2**

| Prevalence | 1-10% | 10-25% | >25% | >75% |
| --- | --- | --- | --- | --- |
|  | Intense itching (F)² | Temporary nasal dryness (N) ² | Temporal hollowing (T)¹ | Temporary swelling^6^ |
|  | Minor hair loss (F)² | Rhinorrhea (N)² | Immediate postoperative headache^5^ | Transient numbness^7^ |
|  | Abnormal intraoperative bleeding (F,T,O,N)² | Slight recurrence of septal deviation (N)² | Alopecia^5^ | Shooting pain^7^ |
|  | Short-term neck stiffness (O)² | Temporary intense itching (F) ¹ | Periorbital ecchymosis^5^ |  |
|  | Epistaxis requiring desmopressin (N)² | Temporary paresthesia^6^ | Temporary ecchymosis^6^ |  |
|  | Sinus infection (N)² |  |  |  |
|  | Long-term neck stiffness (O)² |  |  |  |
|  | Hematoma (F)² |  |  |  |
|  | Unilateral airway reduction (N)² |  |  |  |
|  | Significantly major hair loss (F)² |  |  |  |
|  | Numbness 1 yr postoperatively (T)¹^,7^ |  |  |  |
|  | Uneven brow movement (F)¹ |  |  |  |
|  | Temporary hair loss of thinning (T)¹ |  |  |  |
|  | Residual CSC muscle function (F)¹ |  |  |  |
|  | Neck stiffness 1 yr postoperatively (O)¹ |  |  |  |
|  | Temporal nerve injury (T)³ |  |  |  |
|  | Occasional neck stiffness (O)³ |  |  |  |
|  | Occasional neck weakness (O)³ |  |  |  |
|  | Skin numbness (F)³^,5^ |  |  |  |
|  | Hypersensitivity (F)³ |  |  |  |
|  | Hyposensitivity (F)³ |  |  |  |
|  | Occipital numbness after 1 yr (O)⁴ |  |  |  |
|  | Intense itching⁴ |  |  |  |
|  | hypertrophic scar (O)⁴ |  |  |  |
|  | Temporary hypaestesia^6^ |  |  |  |
|  | Wound heamatoma^7^ |  |  |  |
|  | Temporary seroma (O)^7^ |  |  |  |
|  | Scar tissue (removed)^7^ |  |  |  |

| ¹ Guyuron (2009) |
| --- |
| ² Guyuron (2005) |
| ³ Guyuron (2011) |
| ⁴ Gfrerer (2014)  ^5^ Janis (2011)  ^6^ Jose (2017)  ^7^ Gfrerer (2018) |
